# Potential humoral mediators of remote ischemic preconditioning in patients undergoing surgical coronary revascularization

**DOI:** 10.1038/s41598-017-12833-2

**Published:** 2017-10-04

**Authors:** Nilgün Gedik, Eva Kottenberg, Matthias Thielmann, Ulrich H. Frey, Heinz Jakob, Jürgen Peters, Gerd Heusch, Petra Kleinbongard

**Affiliations:** 1 Institute for Pathophysiology, West German Heart and Vascular Center Essen, Universitätsklinikum Essen, Universität Duisburg- Essen, Essen, Germany; 2 Klinik für Anästhesiologie und Intensivmedizin, Universitätsklinikum Essen, Universität Duisburg-Essen, Essen, Germany; 3 Department of Thoracic and Cardiovascular Surgery, West German Heart and Vascular Center Essen, Universitätsklinikum Essen, Universität Duisburg- Essen, Essen, Germany

## Abstract

Remote ischemic preconditioning (RIPC) by repeated brief cycles of limb ischemia/reperfusion may reduce myocardial ischemia/reperfusion injury and improve patients‘ prognosis after elective coronary artery bypass graft (CABG) surgery. The signal transducer and activator of transcription (STAT)5 activation in left ventricular myocardium is associated with RIPC´s cardioprotection. Cytokines and growth hormones typically activate STATs and could therefore act as humoral transfer factors of RIPC´s cardioprotection. We here determined arterial plasma concentrations of 25 different cytokines, growth hormones, and other factors which have previously been associated with cardioprotection, before (baseline)/after RIPC or placebo (n = 23/23), respectively, and before/after ischemic cardioplegic arrest in CABG patients. RIPC-induced protection was reflected by a 35% reduction of serum troponin I release. With the exception of interleukin-1α, none of the humoral factors changed in their concentrations after RIPC or placebo, respectively. Interleukin-1α, when normalized to baseline, increased after RIPC (280 ± 56%) but not with placebo (97 ± 15%). The interleukin-1α concentration remained increased until after ischemic cardioplegic arrest and was also higher than with placebo in absolute concentrations (25 ± 6 versus 16 ± 3 pg/mL). Only interleukin-1α possibly fulfills the criteria which would be expected from a substance to be released in response to RIPC and to protect the myocardium during ischemic cardioplegic arrest.

## Introduction

Remote ischemic conditioning (RIC) by brief episodes of ischemia/reperfusion in parenchymal organs or limbs before (remote ischemic preconditioning; RIPC) or during (remote ischemic perconditioning) sustained myocardial ischemia and subsequent reperfusion is a non-invasive strategy to protect the myocardium from irreversible ischemia/reperfusion injury. The protection by RIC has been demonstrated in many experimental studies and confirmed in patients undergoing elective interventional^1^ or surgical coronary revascularization^2–5^ and in patients with reperfused acute myocardial infarction^6–10^. The efficacy of RIC was established by a reduction in cardiac biomarker release^1–5,9^ or by cardiac imaging^6–8,10^ and resulted in improved short-term^4,7^ and long-term clinical outcome^1,3,11^. However, two large-scaled randomized trials in patients undergoing cardiac surgery, ERICCA^12^ and RIPHeart^13^, were neutral and did not confirm reduced biomarker release and improved clinical outcome with RIPC. Potential reasons for the lack of protection by RIPC in both trials relate to the use of propofol anesthesia^14,15^ and the inclusion of patients undergoing isolated or additional valve surgery which causes traumatic rather than ischemia/reperfusion myocardial injury and may have diluted the protection by RIPC^15,16^. For a more successful use of RIC in patients, a better understanding of the signal transfer from the stimulus site to the heart and of RIC´s intracellular signal transduction is mandatory.

In different animal models and in healthy volunteers, a neuronal and a humoral signal transfer as well as a neurohumoral interaction in signal transfer have been proposed^17,18^. A humoral signal transfer has been evidenced by the transfer of cardioprotection via plasma^19–22^ or plasma-derived dialysate/filtrate^23–25^ from one individual to another individual’s heart, even across species. In respective experiments, several amino acids^26–29^, cytokines/chemokines^30–33^, neuropeptides^34,35^ as well as other substances, such as adenosine^36,37^, apolipoprotein-A1 (Apo-A1)^38,39^, circulating RNase-1^40^, glucagon like peptide-1 (GLP-1)^41^, microRNA-144^42^ and nitrite^24^ have been identified and proposed as potential humoral transfer factors of RIC. Apo-A1, cytokines, circulating RNase-1, microRNA-144 and nitrite have been reported in healthy volunteers in association with the RIC procedure^24,33,38,40,42^. In patients undergoing cardiac surgery, only some of the potential humoral transfer factors (amino acids, circulating RNase-1, cytokines/chemokines) have been associated with the RIC procedure^26,30,31,40^, but only in two studies there was also a reduction of myocardial injury by RIC^30,31^, and one of these studies was in infants^30^.

Within the myocardium, the putative humoral factors activate intracellular signaling pathways, which ultimately transmit the cardioprotective signal to end-effectors, notably the mitochondria^22,43,44^. Conceptually, the intracellular signaling pathways have been categorized as the nitric oxide synthase/protein kinase G pathway, the reperfusion injury salvage kinase pathway, and the survival activating factor enhancement pathway^18,45,46^. In left ventricular biopsies of patients undergoing coronary artery bypass graft (CABG) surgery, only the phosphorylation of signal transducer and activator of transcription (STAT)5 of the survival activating factor enhancement pathway^47^ was associated with cardioprotection by RIPC^48,49^. STAT is typically activated by members of the cytokine and the growth hormone family^44,50,51^. Therefore, cytokines and growth hormones could potentially serve as humoral transfer factors of RIPC in patients.

We have now quantified the arterial concentration of a number of humoral factors, which may potentially activate STAT and the survival activating factor enhancement pathway, in a cohort of consecutive patients undergoing CABG surgery under isoflurane anesthesia before and after RIPC/placebo, respectively, and before and after ischemic cardioplegic arrest: chemokines/cytokines, i.e. erythropoietin (EPO)^52^, interleukin-(IL-)1α^53^, IL-1β^54^, IL-2^55^, IL-6^56^, IL-8^57^, IL-10^58^, IL-15^55^, IL-17^59^, IL-33^60^, stromal cell-derived factor-1α (SDF-1α)^61^, tumor necrosis factor-α (TNF-α)^62^ and growth hormones, i.e. growth hormone (GH)^63,64^, growth differentiation factor-11 (GDF-11)^65^, growth hormone releasing hormone (GHRH)^66^, growth hormone-releasing peptide (GHRP)^67^. In addition, we determined a few other factors which have been reported before in association with cardioprotection and/or STAT activation, i.e. Apo-A1^38,39^, GLP-1^41^, HIF-1α^68,69^, leptin^70,71^, pentraxin-3^72^, prolactin^73^, RNase-1^40^, survivin^74,75^ and thymosin-β4^76,77^.

## Results

### Cardioprotection by RIPC

**Figure 1:**
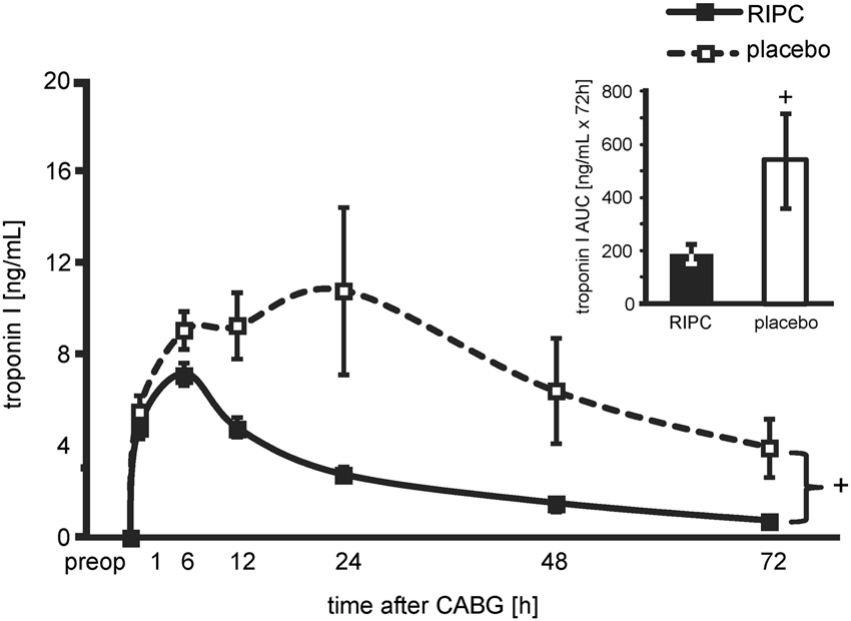
Serum concentration of troponin I. The serum concentration of troponin I at baseline before (preop) and over 72 h after coronary artery bypass graft (CABG) surgery in patients undergoing remote ischemic preconditioning (RIPC; n = 23, black symbols/bars) or placebo (n = 23, white symbols/bars). Decreased troponin I concentrations confirmed protection by RIPC. Insert: area under the curve (AUC) for serum troponin I concentrations over 72 h. ^+^p < 0.05 versus RIPC using 2-way ANOVA for repeated measures or unpaired Student’s t-test (AUC).

Demographics and intraoperative characteristics were not different between patients with RIPC and placebo, respectively (Table 1). The preoperative serum troponin I (TnI) concentration did not differ between patients with RIPC and placebo, respectively. The TnI concentration area under the curve (AUC) over 72 h was decreased by RIPC, indicating cardioprotection (190 ± 16 versus 543 ± 145 ng/mL × 72 h, p = 0.015; Fig. 1). In this small cohort of consecutive patients, the RIPC-related decrease in TnI release was more pronounced than that in the larger cohort reported before^3^.

**Table 1:** Patient demographics and intraoperative characteristics of patients.

	RIPC (n = 23)	placebo (n = 23)	p-value
**demographics**			
age [years]	66.4 ± 1.5	67.7 ± 2.0	0.479
sex [male]	23	19	0.109
body weight [kg]	87.2 ± 2.7	84.6 ± 2.6	0.499
**risk factors and co-morbidities**			
diabetes mellitus	11	6	0.221
hypertension	20	22	0.608
hyperlipidemia	9	8	1.000
peripheral vessel disease	2	4	0.666
COPD	4	2	0.666
renal disease [creatinine > 200 μmol/L]	1	3	0.608
**cardiac status**			
angina CCS III–IV	1	2	1.000
previous myocardial infarction	2	5	0.414
left ventricular ejection fraction [%]	50.5 ± 2.1	51.5 ± 2.3	0.747
**medication**			
aspirin	23	19	0.109
clopidogrel	4	2	0.666
β-blockers	20	16	0.284
statins	17	17	1.000
ACE inhibitors or ARBs	8	10	0.763
**risk scores**			
additive EuroSCORE	3.9 ± 0.5	5.0 ± 0.6	0.174
logistic EuroSCORE [%]	3.4 ± 0.5	5.1 ± 1.0	0.109
EuroSCORE II [%]	1.8 ± 0.2	2.8 ± 0.4	0.058
**intraoperative characteristics**			
time from end of RIPC/placebo to ischemic cardioplegic arrest [min]	64.6 ± 8.0	49.8 ± 10.0	0.280
time from end of RIPC/placebo to reperfusion [min]	130.2 ± 8.1	118.8 ± 7.1	0.304
aortic cross-clamp duration [min]	70.0 ± 4.8	65.4 ± 3.7	0.454
cardioplegia [mL]	1528 ± 46	1546 ± 49	0.798
reperfusion time [min]	34.8 ± 3.1	38.9 ± 3.6	0.393
number of bypass grafts	3.7 ± 0.2	3.6 ± 0.2	0.752
transit time graft flow [mL/min]	87.7 ± 12.3	66.6 ± 9.9	0.204

Data are mean ± standard error of the mean or number. Patient demographics and intraoperative characteristics were compared using unpaired Student’s t-test (continuous data) and 2-tailed Fisher’s exact test (categorical data). Chronic obstructive pulmonary disease (COPD), Canadian cardiovascular society score (CCS), angiotensin-converting enzyme (ACE), angiotensin-II-receptor blockers (ARBs), European system for cardiac operative risk evaluation (EuroSCORE), remote ischemic preconditioning (RIPC). Reperfusion time: time from release of aortic cross-clamp to end of cardiopulmonary bypass.

### Concentration of humoral factors

The concentrations of the analyzed humoral factors were not significantly different between RIPC and placebo at baseline, with the exception of prolactin, which was lower with RIPC than with placebo (Table 2). To normalize for interindividual differences, the concentrations of all factors were also normalized to their baseline.

**Table 2:** Concentration of humoral factors.

parameter	protocol	original data				parameter	protocol	data normalized to baseline		
		baseline	after placebo/RIPC	before ischemic cardioplegic arrest	after ischemic cardioplegic arrest			after placebo/RIPC	before ischemic cardioplegic arrest	after ischemic cardioplegic arrest
Apo-A1 [ng/mL]	RIPC	408 ± 36	365 ± 42	364 ± 36	251 ± 32*^,#^	Apo-A1 [%]	RIPC	106 ± 23	108 ± 18	68 ± 9*^,#^
	placebo	351 ± 35	337 ± 57	292 ± 35	164 ± 21*^,#^		placebo	115 ± 23	94 ± 12	55 ± 10*^,#^
EPO [pg/mL]	RIPC	22 ± 2	22 ± 2	20 ± 2	22 ± 3	EPO [%]	RIPC	134 ± 35	105 ± 26	103 ± 19
	placebo	28±3	26 ± 3	22 ± 3	21 ± 3		placebo	100 ± 15	84 ± 11	77 ± 9
GDF-11 [fg/mL]	RIPC	6836 ± 1544	5678 ± 1031	6104 ± 1174	15244 ± 2244*^,#^	GDF-11 [%]	RIPC	99 ± 17	106 ± 17	275 ± 54*^,#^
	placebo	5314 ± 633	5960 ± 771	6329 ± 852	9764 ± 1303*^,#,+^		placebo	126 ± 19	142 ± 22	219 ± 34*^,#^
GHRH [fg/mL]	RIPC	1694 ± 56	1636 ± 52	1656 ± 48	1621 ± 59	GHRH [%]	RIPC	99 ± 4	100 ± 4	98 ± 4
	placebo	1538 ± 55	1664 ± 59	1699 ± 54	1636 ± 48		placebo	109 ± 2^+^	113 ± 4*^,+^	109 ± 4*^,+^
GHRP [fg/mL]	RIPC	998 ± 189	1008 ± 208	1220 ± 236	1096 ± 187	GHRP [%]	RIPC	275 ± 180	338 ± 187	123 ± 18
	placebo	1211 ± 263	1147 ± 240	1120 ± 236	1117 ± 145		placebo	120 ± 16	137 ± 18	225 ± 37
GLP-1 [pg/mL]	RIPC	1.5 ± 0.2	1.4 ± 0.2	1.5 ± 0.2	2.2 ± 0.2*^,#^	GLP-1 [%]	RIPC	93 ± 3	103 ± 7	173 ± 17*^,#^
	placebo	1.5 ± 0.2	1.5 ± 0.2	1.6 ± 0.2	2.4 ± 0.2*^,#^		placebo	107 ± 8	122 ± 16	201 ± 29*^,#^
GH [pg/mL]	RIPC	830 ± 146	323 ± 85*	221 ± 44*	882 ± 158^#^	GH [%]	RIPC	60 ± 19	153 ± 99	500 ± 327*
	placebo	510 ± 125	401 ± 124	320 ± 93	1059 ± 270*^,#^		placebo	127 ± 33	305 ± 116	820 ± 221*^,#^
HIF-1α [fg/mL]	RIPC	18 ± 2	16 ± 3	17 ± 2	17 ± 2	HIF-1α [%]	RIPC	101 ± 20	102 ± 12	101 ± 10
	placebo	19 ± 3	21 ± 5	20 ± 5	22 ± 6		placebo	133 ± 17	130 ± 18	159 ± 33
IL-1α [pg/mL]	RIPC	12 ± 2	20 ± 2	16 ± 2	25 ± 6*^,#^	IL-1α [%]	RIPC	280 ± 56*	235 ± 96*	298 ± 71*
	placebo	18 ± 3	18 ± 2	15 ± 3	16 ± 3^+^		placebo	97 ± 15^+^	97 ± 16^+^	135 ± 40^+^
IL-1β [fg/mL]	RIPC	746 ± 220	734 ± 210	881 ± 223	1630 ± 316*^,#^	IL-1β [%]	RIPC	220 ± 94	251 ± 75	517 ± 173*^,#^
	placebo	631 ± 97	752 ± 100	739 ± 103	1367 ± 222*^,#^		placebo	169 ± 28	178 ± 46	337 ± 66*
IL-2 [fg/mL]	RIPC	4936 ± 440	6205 ± 735	5391 ± 601	5908 ± 685	IL-2 [%]	RIPC	143 ± 27	160 ± 44	189 ± 53*
	placebo	7040 ± 1326	7291 ± 1000	5114 ± 458	7351 ± 1098		placebo	132 ± 21	103 ± 14	211 ± 59*^,#^
IL-6 [fg/mL]	RIPC	4108 ± 715	4133 ± 664	5498 ± 947	14633 ± 941*^,#^	IL-6 [%]	RIPC	105 ± 6	166 ± 23	588 ± 92*^,#^
	placebo	6239 ± 882	6169 ± 890	6251 ± 789	16572 ± 1200*^,#^		placebo	99 ± 2	129 ± 16	439 ± 85*^,#,+^
IL-8 [pg/mL]	RIPC	14 ± 1	14 ± 1	18 ± 3	59 ± 12*^,#^	IL-8 [%]	RIPC	100 ± 2	125 ± 22	441 ± 65*^,#^
	placebo	16 ± 2	15 ± 2	18 ± 3	49 ± 9*^,#,+^		placebo	100 ± 4	122 ± 20	367 ± 42*^,#^
IL-10 [fg/mL]	RIPC	3449 ± 826	3943 ± 1034	11320 ± 5458	56674 ± 1921*^,#^	IL-10 [%]	RIPC	107 ± 7	324 ± 129	2227 ± 792*^,#^
	placebo	2875 ± 419	3096 ± 487	3827 ± 884	57356 ± 1216*^,#^		placebo	109 ± 11	146 ± 23	3120 ± 1007*^,#^
IL-15 [fg/mL]	RIPC	4380 ± 280	4312 ± 324	3933 ± 258	4832 ± 300^#^	IL-15 [%]	RIPC	102 ± 6	93 ± 6	117 ± 9^#^
	placebo	5253 ± 690	5633 ± 566	5218 ± 682	6234 ± 613*^,#^		placebo	120 ± 14	108 ± 14	134 ± 19*^,#^
IL-17 [pg/mL]	RIPC	20 ± 2	28 ± 2	32 ± 4*	35 ± 5*	IL-17 [%]	RIPC	174 ± 24	212 ± 51*	266 ± 69*
	placebo	28 ± 3	29 ± 4	32 ± 7	36 ± 9		placebo	120 ± 18	103 ± 18^+^	143 ± 32^+^
IL-33 [fg/mL]	RIPC	3997 ± 544	5178 ± 525	5983 ± 665	19054 ± 1826*^,#^	IL-33 [%]	RIPC	146 ± 15	178 ± 23	615 ± 92*^,#^
	placebo	3679 ± 530	5454 ± 675	7059 ± 1587*	21732 ± 1888*^,#^		placebo	153 ± 13	190 ± 40	633 ± 72*^,#^
leptin [pg/mL]	RIPC	56 ± 9	49 ± 9	43 ± 8	40 ± 1	leptin [%]	RIPC	88 ± 3*	74 ± 3*	67 ± 3*^,#^
	placebo	73 ± 23	65 ± 23	50 ± 11*	48 ± 8*		placebo	86 ± 3*	77 ± 3*	72 ± 4*
pentraxin-3 [pg/mL]	RIPC	804 ± 137	808 ± 129	1002 ± 1211	3453 ± 281*^,#^	pentraxin-3 [%]	RIPC	106 ± 4	160 ± 16	697 ± 104*^,#^
	placebo	1061 ± 204	982 ± 160	1240 ± 162	4745 ± 831*^,#,+^		placebo	99 ± 5	159 ± 23	928 ± 239*^,#^
prolactin [ng/mL]	RIPC	34 ± 3	44 ± 4	52 ± 5*	58 ± 7*	prolactin [%]	RIPC	158 ± 31	208 ± 56*	233 ± 51*
	placebo	48 ± 4^+^	60 ± 6^+^	61 ± 7	52 ± 7		placebo	138 ± 30	143 ± 35	119 ± 29^+^
RNase-1 [pg/mL]	RIPC	663 ± 150	464 ± 94	728 ± 100	1744 ± 120*^,#^	RNase-1 [%]	RIPC	93 ± 10	200 ± 37	405 ± 69*^,#^
	placebo	477 ± 37	460 ± 82	795 ± 94*	1627 ± 241*^,#^		placebo	131 ± 20	244 ± 33*	519 ± 96*^,#^
SDF-1α [pg/mL]	RIPC	2270 ± 94	2197 ± 99	2766 ± 126*	2846 ± 98*	SDF-1α [%]	RIPC	97 ± 1	123 ± 4*	127 ± 3*
	placebo	2382 ± 102	2327 ± 97	2881 ± 105*	2922 ± 120*		placebo	98 ± 2	124 ± 5*	126 ± 5*
surviving [pg/mL]	RIPC	36 ± 9	46 ± 7	34 ± 3	58 ± 1	surviving [%]	RIPC	227 ± 51	152 ± 25	313 ± 57
	placebo	45 ± 7	56 ± 9	51 ± 8	90 ± 8*^,#,+^		placebo	151 ± 28	193 ± 56	472 ± 277*^,#^
thymosin-β4 [ng/mL]	RIPC	349 ± 29	322 ± 25	285 ± 22	280 ± 31	thymosin-β4 [%]	RIPC	96 ± 4	90 ± 7	99 ± 15
	placebo	364 ± 46	371 ± 39	362 ± 42	316 ± 27		placebo	110 ± 9	109 ± 8	105 ± 11
TNF-α [fg/mL]	RIPC	2973 ± 744	3107 ± 737	3259 ± 800	4301 ± 744*^,#^	TNF-α [%]	RIPC	108 ± 3	116 ± 6	198 ± 42*^,#^
	placebo	2892 ± 580	3105 ± 566	2772 ± 265	3827 ± 450*^,#^		placebo	111 ± 3	116 ± 7	166 ± 19*^,#^

Data are mean  ±  standard error of the mean. Concentrations of all humoral factors were analyzed by 2-way (group, time) ANOVA for repeated measures followed by Fisher’s post hoc tests. *p < 0.05 versus baseline, ^#^p < 0.05 versus before ischemic cardioplegic arrest, ^+^p < 0.05 versus RIPC. Apolipoprotein A1 (Apo-A1), erythropoietin (EPO), growth differentiation factor-11 (GDF-11), growth hormone (GH), growth hormone-releasing peptide (GHRP), glucagon like peptide-1 (GLP-1), hypoxia inducible factor 1α (HIF-1α), interleukin (IL), remote ischemic preconditioning (RIPC), ribonuclease A (RNase-1), stromal cell derived factor-1 α (SDF-1α), tumor necrosis factor-α (TNF-α).

The concentrations (normalized and not normalized to baseline) of Apo-A1, EPO, GHRP, GLP-1, GH, HIF-1α, IL-1β, IL-2, IL-10, IL-15, IL-33, leptin, RNase-1, SDF-1α, thymosin-β4 and TNF-α did not differ between RIPC and placebo at all analyzed time points (Table 2). The concentration of Apo-A1 decreased, whereas the concentrations of GLP-1, GH, IL-1β, IL-10, IL-15, IL-33, RNase-1, SDF-1α and TNF-α increased after ischemic cardioplegic arrest over that at baseline and before ischemic cardioplegic arrest (Table 2).

**Figure 2:**
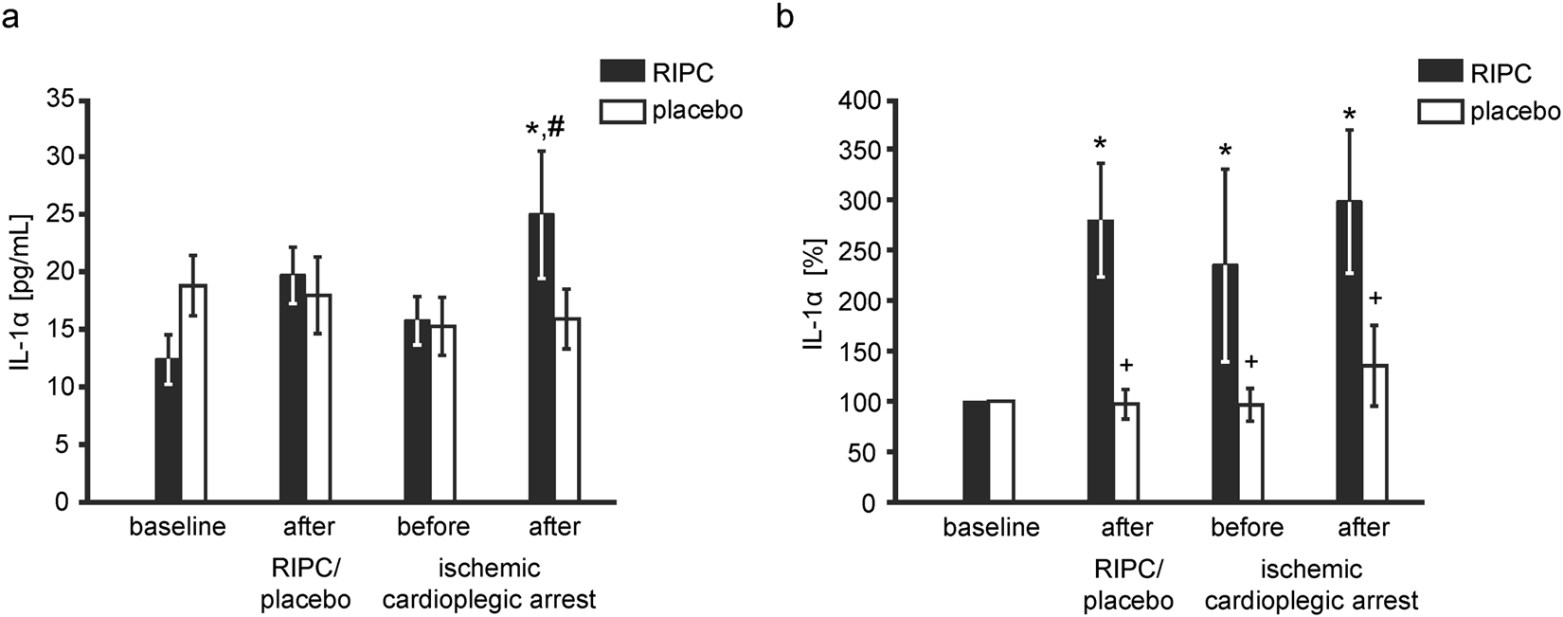
Plasma concentration of interleukin-1α. The plasma concentration of interleukin-1α (IL-1α) before (baseline) and after remote ischemic preconditioning (RIPC; n = 23, black bars) or the placebo protocol (n = 23, white bars) and before and after ischemic cardioplegic arrest, respectively, in patients undergoing coronary artery bypass graft surgery. The plasma concentration of IL-1α was increased after ischemic cardioplegic arrest with RIPC and was greater with RIPC than with placebo (**a**). After normalization to baseline, the IL-1α plasma concentration was greater with RIPC than with placebo throughout the remaining protocol (**b**). *p < 0.05 versus baseline, ^#^p < 0.05 versus before ischemic cardioplegic arrest, ^+^p < 0.05 versus RIPC using 2-way ANOVA for repeated measures, followed by Fisher’s post hoc tests.

The IL-1α concentration, when normalized to baseline, increased after the RIPC procedure and remained increased until after ischemic cardioplegic arrest, whereas it was unchanged with placebo. In absolute concentrations, interleukin-1α increased after ischemic cardioplegic arrest over that at baseline and before ischemic cardioplegic arrest with RIPC, whereas it did not change over time with placebo (Table 2 and Fig. 2).

The concentrations of GDF-11 and IL-8 increased after ischemic cardioplegic arrest and were greater with RIPC than with placebo, but after normalization to baseline these changes were no longer significant (Table 2). The concentrations of pentraxin-3 and survivin increased after ischemic cardioplegic arrest and were lower with RIPC than with placebo, but again after normalization to baseline these changes were no longer significant (Table 2).

Exclusively after normalization to baseline, the GHRH concentration was lower with RIPC than with placebo throughout the remaining protocol. The normalized concentrations of IL-6 and prolactin were greater with RIPC than with placebo after ischemic cardioplegic arrest. The normalized concentration of IL-17 was greater with RIPC than with placebo before and after ischemic cardioplegic arrest (Table 2).

## Discussion

Except for IL-1α, none of the analyzed humoral factors in our study appeared to fulfill the criteria for a transfer factor of cardioprotection by RIC (increase in the factor’s concentration after the RIC procedure and before myocardial ischemia as well as association with reduced myocardial ischemia/reperfusion injury), and we thus add another mostly negative study to the so far elusive search for RIC´s transfer factor^17^. Our study was unique in that it was conducted in patients undergoing CABG surgery, where the RIPC procedure indeed induced cardioprotection. However, none of the humoral factors differed in absolute concentration between RIPC and placebo before ischemic cardioplegic arrest. The concentrations of some factors (GDF-11, GHRH, IL-1α, IL-6, IL-8 and IL-17) were greater with RIPC than with placebo after ischemic cardioplegic arrest. For these factors, however, it is unclear whether this difference is truly related to myocardial ischemia/reperfusion injury and protection from it. Cardiopulmonary bypass inflicts a systemic inflammatory injury to the entire body and induces damage to various parenchymal organs^78^. RIC, in turn, is also a systemic response and provides protection to a number of parenchymal organs^79,80^. Therefore, the observed differences in the concentrations of the above humoral factors may originate from other organs than the heart.

The IL-1α concentration, when normalized to baseline, was increased after the RIPC procedure and it remained increased until after ischemic cardioplegic arrest whereas it was not changed throughout the placebo protocol. In absolute concentrations, IL-1α was also greater with RIPC than with placebo after ischemic cardioplegic arrest. IL-1α is a member of the IL-1 cytokine family and involved in inflammatory processes. IL-1α is released from macrophages, monocytes, endothelial and epithelial cells^81,82^ but also from cardiomyocytes^83^ in response to cell injury. In mice with myocardial infarction, IL-1α was released into the systemic circulation, whereas IL-1α in the myocardial tissue did not change^83^. In isolated perfused rat hearts, IL-1α blockade after reperfusion reduced infarct size^84^, suggesting that intracellular IL-1α contributes to ischemia/reperfusion injury. However, exogenous IL-1α preconditioning^85^ and pretreatment^86^ in isolated perfused rat hearts improved ventricular systolic pressure and reduced infarct size, suggesting that circulating, extracellular IL-1α induces cardioprotection. A causal role of IL-1α as humoral mediator and trigger for intracellular signaling in RIC remains to be established. Whereas IL-1β is known to activate STATs^54^, the exact role of IL-1α in STAT activation is not clear so far. IL-1α could indirectly activate STATs by induction of IL-6^53^. Except for IL-1α, which has not been associated with RIC before, we could not confirm any of the previously reported humoral factors to be associated with cardioprotection by RIC.

There are limitations of our current study: 1) Given our small patient cohort and the high number of analyzed humoral factors, the risk of a type I error with respect to IL-1α is high, in particular since its increase after the RIPC procedure was only evident with normalized data. Our exploratory study is hypothesis generating, so replication in a larger cohort of patients is mandatory. 2) We used plasma samples from a consecutive patient cohort with co-morbidities and co-medications, some of which may potentially interfere with the protection by ischemic conditioning maneuvers^87–89^, but also with the release of humoral factors. Patients undergoing RIPC were younger and had lower preoperative risk scores than those undergoing the placebo procedure, and these differences may have contributed to the more pronounced decrease in TnI release than that in the larger cohort reported before^3^. 3) We analyzed the plasma concentrations only at four defined time points, i.e. before/5 min after the RIPC/placebo protocol and before/10 min after ischemic cardioplegic arrest, not considering for the potentially different kinetics of each humoral factor. In particular, the time from the end of the RIPC/placebo procedure to ischemic cardioplegic arrest was a bit longer in patients with RIPC than with placebo, and we may have missed a transient increase or decrease in humoral factors with RIPC.

## Methods

### Ethics Statement

The study conforms to the principles of the Declaration of Helsinki. With approval by the local ethics committee (Institutional Review Board, University of Duisburg-Essen, no. 08–3683) and patients’ written informed consent, arterial blood samples were harvested from a small cohort of consecutive patients (n = 23 RIPC/23 placebo) who underwent elective isolated first-time CABG surgery^3^. These patients were enrolled between February 2012 and April 2013 and within the framework of a larger, randomized, prospective, double-blind, placebo-controlled trial (ClinicalTrials.gov NCT01406678, date of registration: December 1, 2009). The inclusion and exclusion criteria for the trial as well as its results have been reported^3^.

### Study procedure

Anesthesia was induced with sufentanil (1 µg/kg), etomidate (0.3 mg/kg) and rocuronium (0.6 mg/kg) and maintained with isoflurane (0.6–1.0% end-tidal). The RIPC protocol consisted of 3 cycles of 5 min left upper arm ischemia/5 min reperfusion, and data were compared to placebo (cuff left deflated for 30 min). CABG was performed using median sternotomy, mild systemic hypothermia (>32 °C) and antegrade cold crystalloid Bretschneider (Köhler Chemie GmbH, Bensheim, Germany) cardioplegia, with additional topical cooling and single aortic cross-clamping for all distal anastomoses^3^.

### Arterial blood samples and plasma preparation

Arterial blood samples were taken before (baseline) and 5 min after the end of the RIPC/placebo procedure as well as before and 10 min after the ischemic cardioplegic arrest. These time points were chosen to detect changes induced by the RIPC protocol per se and the interaction of RIPC with ischemic cardioplegic arrest. At each time point, 25 mL arterial blood was withdrawn and sampled in vials containing lithium-heparin (Sarstedt, Nümbrecht, Germany). The arterial blood was then immediately centrifuged at 4 °C with 800 g for 15 min, plasma was separated, stored at −80 °C for later use and again centrifuged for 10 min at 4500 g before use. Additionally, 5 mL of arterial blood was withdrawn in separate vials (Sarstedt, Nümbrecht, Germany) to analyze the serum concentration of prolactin.

### Serum troponin I

Venous blood samples were withdrawn from each patient on the day before surgery and postoperatively at 1, 6, 12, 24, 48, and 72 h. Serum TnI concentration was measured using a specific two-side immunoassay with the DimensionR RxL MaxR integrated system (Dimension Flex, Dade Behring GmbH, Marburg, Germany) in the central laboratory of the University Duisburg-Essen Medical School. The detection range of TnI was 0.04 to 40 µg/L, the upper limit of normal 0.1 µg/L. The AUC for serum TnI concentration was calculated according to the trapezoidal rule. Missing values were replaced by linear inter- and extrapolation^3^.

### Plasma concentrations of humoral factors

The plasma concentrations of humoral factors were determined using enzyme immunoassays. Standards and samples were added to microplates, which were precoated with the specific antibody against the respective protein.

For the detection of Apo-A1^90^, EPO^91^, GDF-11^92^, RNase-1^93^ (ELISA Cloud-Immunoassay, Houston, USA) and HIF-1α^94^ (RayBio, Georgia, USA) avidin-conjugated horseradish peroxidase was supplemented. For the detection of GLP-1^95^ (Abcam, Cambridge, UK) an antibody cocktail consisting of a capture and a detector antibody was supplemented. For the detection of GH^96^, IL-1α^97^, IL-2^98^, IL-15^99^, IL-17^99^, IL-33^100^, leptin^101^, pentraxin-3^102^, SDF-1α^103^ and survivin^104^ (R&D systems, Abingdon, UK) an enzyme-linked polyclonal horseradish peroxidase-conjugated antibody was supplemented. For the detection of GHRH^105^ and GHRP^106^ (ELISA Cloud-Immunoassay, Houston, USA) biotin-conjugated antibodies against the respective protein were added to the microplate, and the antibodies on the plate and the biotin-labeled antibodies then competed for each other. An avidin-conjugated horseradish peroxidase-conjugated secondary antibody was supplemented. For the detection of thymosin-β4^107^ (Immundiagnostik, Bensheim, Germany) an antibody against thymosin-β4 was added to the microplate, which was precoated with the immobilized antigen to thymosin-β4. The antigen of the sample and the immobilized antigen then competed for each other. A horseradish peroxidase-conjugated secondary antibody was supplemented.

After adding the respective substrate, the enzyme-substrate reaction resulted in a blue product. The color intensity was proportional to the concentration of the protein. The reaction was stopped, and the color changed to yellow. The color intensity was measured at 450 nm using a spectrophotometer (Microplate Reader 680, BIORAD, München, Germany).

For the detection of IL-1β^97^, IL-6^108^, IL-8^108^, IL-10^98^ and TNF-α^108^ (R&D systems, Abingdon, UK) an enzyme-linked polyclonal antibody and a substrate solution were supplemented. After adding an amplifier enzyme the enzyme-substrate reaction resulted in a violet product. The color intensity was proportional to the enzyme activity, which was related to the concentration of bound proteins. The reaction was stopped, and the color intensity was measured at 490 nm using a spectrophotometer (Microplate Reader 680, BIORAD, München, Germany).

The prolactin concentration was measured in the central laboratory of the University Duisburg-Essen Medical School. The detection range of prolactin assay was 0.3 μg/L to 200 μg/L. The serum concentration of prolactin was measured using a two-side sandwich chemiluminescence immunoassay with an acridinium ester-conjugated antibody against prolactin and a secondary antibody covalently coupled to paramagnetic particles (ADVIAR Centaur XP, Siemens, Tarrytown, USA)^109^.

The concentrations of the respective proteins were quantified by comparison to a standard curve.

### Statistics

Data are expressed as mean ± standard error of the mean (SEM). Statistics were performed using SigmaStat software (SigmaStat 2.03, SPSS Inc., Chicago, IL, USA). Patient demographics and intraoperative characteristics were compared using unpaired Student’s t-test (continuous data) and 2-tailed Fisher’s exact test (categorical data). Serum TnI of patients was analyzed by 2-way (group, time) ANOVA for repeated measures. The AUC for the serum TnI over 72 h was compared between RIPC and placebo by unpaired Student’s t-test. Plasma concentrations of all humoral factors were analyzed by 2-way (group, time) ANOVA for repeated measures. When a significant difference was detected, ANOVA was followed by Fisher’s post hoc tests. Differences were considered significant at the level of p < 0.05.
